# Disease characteristics, treatment, and outcomes in Chinese chronic lymphocytic leukemia patients following BTK inhibitor discontinuation: a multicenter real-world study

**DOI:** 10.3389/fmed.2026.1739102

**Published:** 2026-06-12

**Authors:** Yuting Yan, Wei Wang, Xiaojing Yan, Hong Xu, Danyang Wu, Tingyu Wang, Wei Liu, Wenyang Huang, Gang An, Dehui Zou, Jiahuang Lin, Yaqun Fu, Lugui Qiu, Shuhua Yi

**Affiliations:** 1 State Key Laboratory of Experimental Hematology, National Clinical Research Center for Blood Diseases, Haihe Laboratory of Cell Ecosystem, Institute of Hematology & Blood Diseases Hospital, Chinese Academy of Medical Sciences & Peking Union Medical College, Tianjin, China; 2 Tianjin Institutes of Health Science, Tianjin, China; 3 Department of Haematology, The Affiliated Hospital of Qingdao University, Qingdao, China; 4 Department of Haematology, First Affiliated Hospital of China Medical University, Shenyang, China; 5 Epidemiology Asia Pacific Unit, Biostatistics and Research Decision Sciences (BARDS), MSD R&D (China) Co., Ltd, Shanghai, China

**Keywords:** Bruton tyrosine kinase inhibitor, chronic lymphocytic leukemia, clinical outcome, discontinuation, small lymphocytic lymphoma

## Abstract

Utilization of novel Bruton tyrosine kinase inhibitors (BTKi) has become common for treating CLL/SLL patients, yet limited evidence exists on the clinical characteristics and outcomes following BTKi therapy discontinuation in China. This multicenter retrospective study analyzed 37 CLL/SLL patients in China who discontinued BTKi therapy. The mean age at discontinuation was 62.67 years, with the majority being male (67.57%). Most patients (62.16%) were relapsed/refractory (R/R) CLL/SLL patients prior to BTKi treatment, and 37.84% were treatment-naïve patients. Treatment-naïve patients were significantly younger than R/R patients (56.93 vs. 66.16 years, *p* = 0.005). Discontinuation reasons included resistance (48.65%), intolerance (32.43%), and other factors (18.92%). The most frequently used regimen among the post-BTKi first subsequent therapies was the anti-CD20 antibody combination therapy (52.90%). The overall disease control rate during BTKi treatment was 78.13%. The median progression-free survival (PFS) for BTKi therapy was 19.09 months, 19.09 months for treatment-naïve patients, and 14.39 months for R/R patients. The minimum median PFS was observed in patients with resistance (7.86 months). After BTKi discontinuation, median PFS was shorter: 8.87 months for first subsequent therapy and 5.32 months for second subsequent therapy. No significant difference was observed in overall survival. These findings illustrate the impact of prior treatment and discontinuation reasons on subsequent outcomes.

## 1 Introduction

Chronic lymphocytic leukemia (CLL) and small lymphocytic lymphoma (SLL) are characterized by progressive accumulation of leukemic cells in blood, bone marrow, and lymphoid tissues (1). They are different manifestations of the same disease and share similar treatment approaches (1). CLL/SLL is predominantly affecting middle-aged and elderly individuals (2). Global incidence rates of CLL/SLL have been rising in recent decades (3), with approximately 191,000 new cases and 61,000 deaths reported annually (4). In western countries, CLL/SLL is the most prevalent hematologic malignancy, with an annual incidence rate of 4.2/100,000 in the general population and exceeding 30/100,000 in individuals over 80 years of age (5). Although the incidence of CLL/SLL in China is relatively low, accounting for approximately 1% to 3% of Non-Hodgkin's Lymphoma (6), there has been a rapid increase attributed to population aging and expanded cancer screening (7). Chinese CLL/SLL patients have been reported to exhibit slightly prolonged overall survival (OS) compared to the US population (8).

The decision to initiate treatment for CLL/SLL relies on patient symptoms and the risk of disease progression (9). Patients deemed to require treatment are categorized as active CLL/SLL patients. Despite approximately 70% to 80% of CLL/SLL patients being asymptomatic at the time of diagnosis, a significant proportion (approximately two-thirds) eventually require treatment (10). Prognosis varies considerably among patients, and while allogeneic stem cell transplantation offers potential curative benefits, the disease is largely considered incurable (11). First-line therapeutic recommendations are influenced by factors such as TP53 status, overall physical condition, and comorbidities (11). Targeted therapies, such as Bruton tyrosine kinase inhibitors (BTKi), often represent the preferred treatment options for CLL/SLL patients harboring del(17p)/TP53 mutations or those experiencing relapse within 3 years (12). The emergence of kinase inhibitors has ushered in a transformative era in CLL/SLL treatment, replacing traditional chemotherapy and chemoimmunotherapy approaches (13). Notably, ibrutinib, an esteemed BTKi, has exhibited notable efficacy in both relapsed/refractory (R/R) and treatment-naïve CLL/SLL patients, making it a widely adopted first-line and second-line treatment option, since its approval in 2014 in the United States and Europe (14). Ibrutinib was approved for CLL/SLL patients with prior therapies in China in 2017, followed by the approvals of zanubrutinib and orelabrutinib in 2020 (15, 16).

Some investigations into patients with CLL/SLL reveals a substantial proportion discontinuing treatment owing to disease progression, drug resistance, adverse reactions, or other factors (17–21). Notably, the discontinuation rates attributed to adverse events (AEs) demonstrate similarity between patients undergoing first-line therapy and those with R/R CLL/SLL (17, 21). Furthermore, patients grappling with such complications and undergoing concurrent treatments exhibit an elevated discontinuation rate due to the utilization of additional agents, such as anticoagulants, antiplatelet drugs, antiarrhythmic drugs, etc. (17). Despite the remarkable efficacy of novel treatments such as ibrutinib and other BTKi in CLL/SLL, several challenges persist in clinical practice. These challenges encompass toxicity, the emergence of resistance, and the requirement for lifelong therapy. However, due to the relatively late approval of BTK inhibitors in China, the availability of long-term follow-up evidence for ibrutinib and other BTKi in real-world settings remains extremely limited. Limited information exists regarding clinical outcomes and the likelihood of therapy disruption or discontinuation outside the controlled environment of clinical trials (13, 22–25), and this is especially true for the Chinese population, where related information is also limited even within the context of clinical trials. Notably, the interruptions to continuous therapy, often necessary to manage treatment-related adverse effects, are more prevalent in real-world settings compared to clinical trials. These interruptions are likely to have a detrimental impact on disease progression rates and final clinical outcomes (26).

Patients involved in clinical trials are generally carefully selected, making it challenging to fully represent the treatment discontinuation of the real world. Therefore, the results from real-world studies become increasingly crucial, especially for the use of new drugs in these diseases. To address this knowledge gap, it is necessary to investigate the clinical profiles of Chinese CLL/SLL patients who discontinued BTKi therapy, thereby addressing the lack of real-world evidence in China. This study aims to comprehensively describe the demographic and clinical features, as well as treatment patterns, of Chinese patients with active CLL/SLL who discontinued BTKi therapy during and after BTKi treatment. Through a thorough analysis of patient characteristics and treatment outcomes, our findings intend to provide valuable insights into the utilization and challenges associated with BTKi therapies in the Chinese CLL/SLL population. By generating comprehensive real-world evidence, we can optimize treatment strategies and enhance the overall management of CLL/SLL patients in China.

## 2 Materials and methods

### 2.1 Data source

This retrospective, multicenter, observational database study used structured secondary data. We identified Chinese adult patients with CLL/SLL who underwent at least one BTKi-related treatment regimen and discontinued BTKi therapy at three Grade-A Tertiary hospitals in Northern China—namely, the Institute of Hematology and Blood Diseases Hospital in Tianjin, The Affiliated Hospital of Qingdao University in Shandong, and The First Hospital of China Medical University in Liaoning—between July 1, 2017, and June 30, 2021, with an observation period extending until December 31, 2021. These hospitals, ranked among the top 100 medical institutions in China, provided comprehensive inpatient and outpatient data extracted from diverse information systems, including Electronic Medical Records (EMR), Hospital Information System, Laboratory Information Management System, Radiology Information System, and Picture Archiving and Communication System. To protect patient privacy, personal information was de-identified, and each patient was assigned an independent study identification number. Ethical approval for the study was obtained from the Ethics Review Committee at each study site.

### 2.2 Inclusion and exclusion criteria

Consistent inclusion criteria were applied to all datasets used in this study. The criteria for inclusion were as follows: (1) patients with a confirmed diagnosis of CLL/SLL or ICD-10 codes of C91.1; (2) patients aged 18 years or older at the time of diagnosis; and (3) patients who received at least one dose of BTKi and discontinued BTKi therapy between July 1, 2017, and June 30, 2021. The following exclusion criterion was applied: patients who experienced Richter's transformation at any time before the time of BTKi discontinuation.

**Figure 1:**
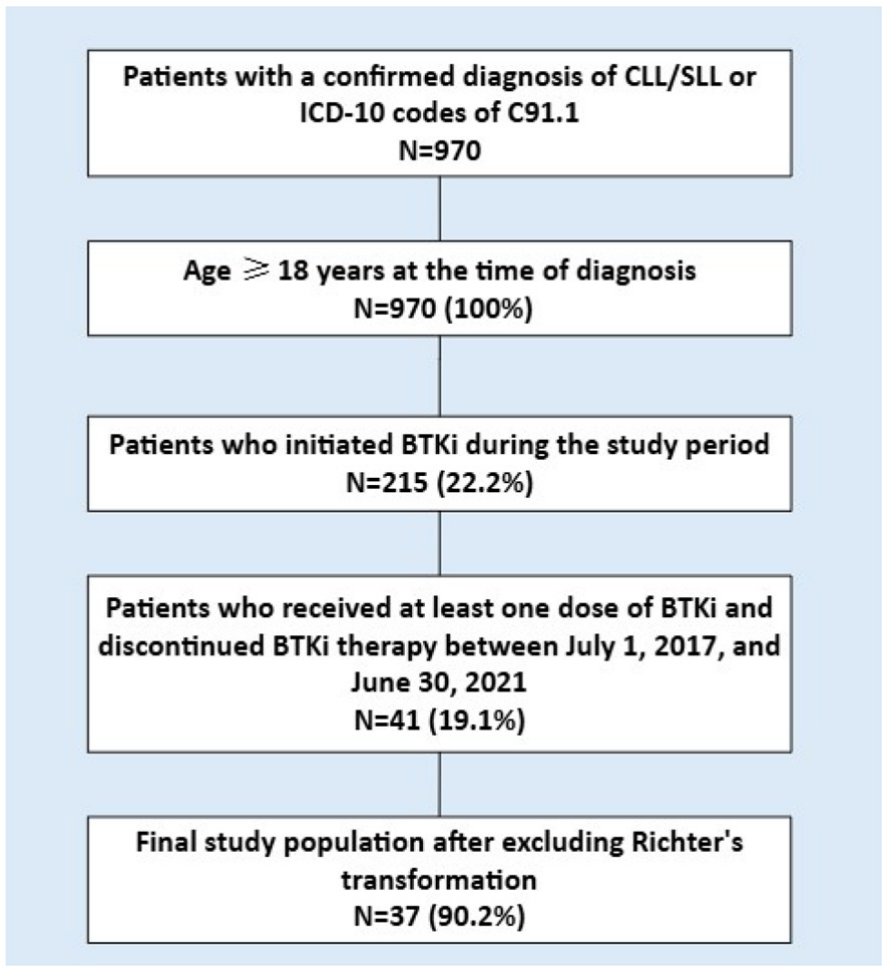
Patient attrition flow chart.

A total of 970 patients with confirmed CLL/SLL were initially extracted from three study sites. Among the 970 CLL/SLL patients, 215 initiated BTKi; among BTKi initiators, 41 discontinued BTKi during the study window, and 37 patients were included in the final analysis (Figure 1).

### 2.3 Study population and subpopulations

The study population comprised Chinese adult patients with active CLL/SLL, categorized into two subpopulations (treatment-naïve and R/R before BTKi treatment) based on their therapy history. Treatment-naïve was defined as patients who received BTKi as initial therapy when enrolled into the study. Major reasons for discontinuation of BTKi therapies were classified as (1) Resistance to BTKi therapies; (2) Intolerance to BTKi therapies; and (3) Discontinuation for other reasons. Resistance to BTKi therapies was defined as patients discontinuing due to progressive diseases (PD) or Richter's transformations (RT) to either diffuse large B-cell lymphoma or Hodgkin lymphoma. Intolerance to BTKi therapies was defined as patients discontinuing due to toxicity related to BTKi therapies. Other reasons included discontinuation due to physician or patient preference, financial concerns, and unspecified reasons.

### 2.4 Study indicators

Demographics, clinical characteristics, and clinical outcomes were summarized for participants. Baseline demographic and clinicopathological variables were collected at the time of BTKi discontinuation, including age, gender, weight, height, alcohol use, smoking history, comorbidities, medical insurance, and other relevant clinical parameters. Treatment patterns were documented for each participant during BTKi therapy and after treatment discontinuation, both in treatment-naïve and R/R CLL/SLL patients. Disease control rate (DCR), progression-free survival (PFS), and OS were calculated to compare the clinical outcomes during BTKi therapy and post-BTKi subsequent therapy for the overall population and subpopulations.

### 2.5 Statistical analysis

Descriptive statistics were presented for patient demographics and clinical characteristics. Categorical variables, such as gender and comorbidities/complications, were summarized by counts and percentages (%), while continuous variables, such as age, were described as means with standard deviations or medians with interquartile ranges (IQRs) as appropriate. Characteristics and treatment patterns of CLL/SLL patients, both treatment-naïve and R/R, who discontinued BTKi, were summarized separately. Two-sided Fisher's exact test or chi-squared test was used for comparing categorical parameters, and Student's *t*-test or Mann-Whitney U-test was employed for examining differences between two continuous variables. The number of events and censoring for DCR and PFS was reported as counts and percentages. The duration from BTKi initiation to discontinuation, PFS and OS was assessed using the Kaplan-Meier method. Statistical analysis was performed using R 4.2.1 statistical software.

## 3 Results

### 3.1 Demographic and clinical characteristics

**Figure 2:**
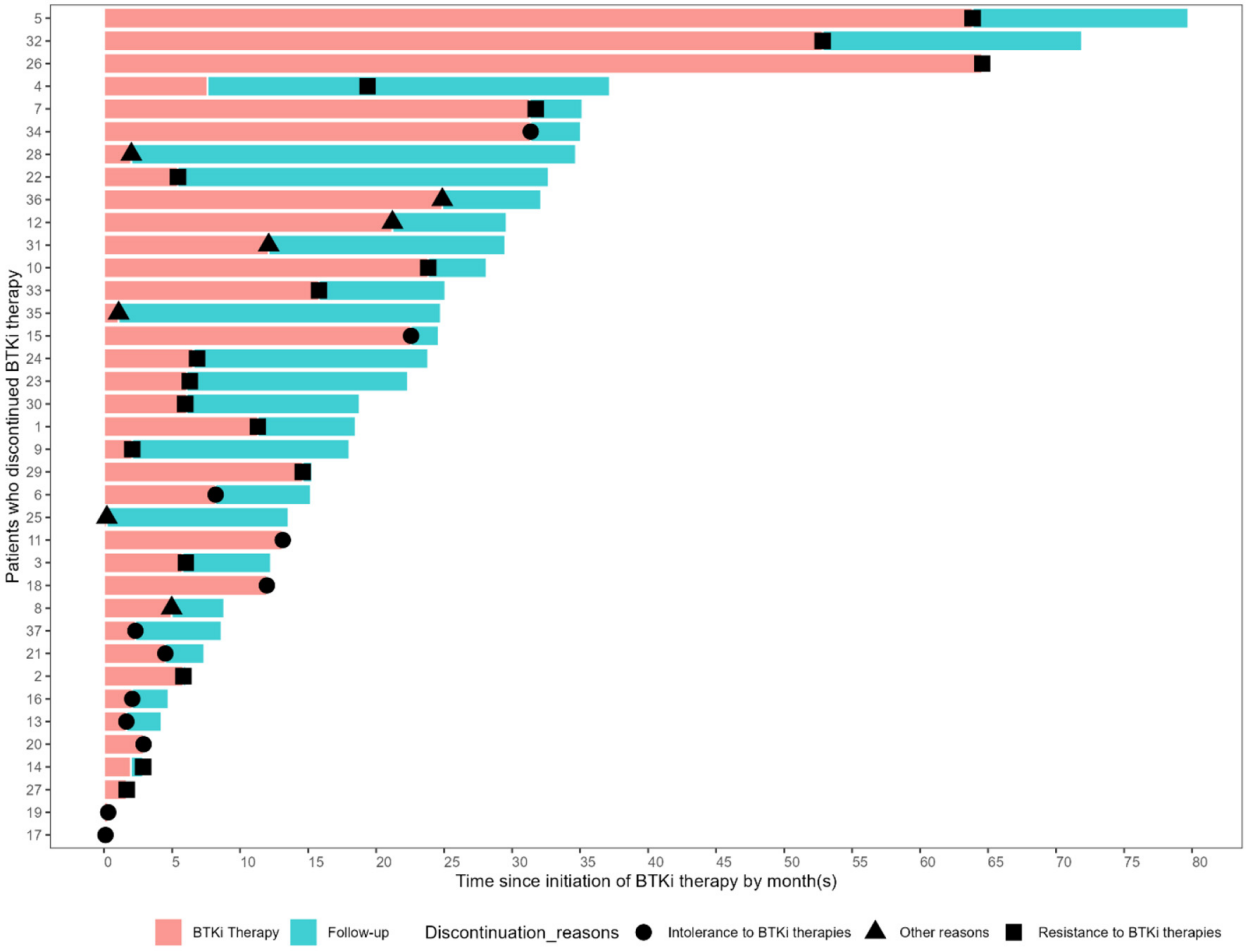
Swimmer plot illustrates reasons for discontinuation in 37 patients who discontinued BTKi therapy.

A total of 37 patients who discontinued BTKi therapy were included in this study (Figure 1). The mean age at the time of BTKi discontinuation was 62.67 ± 10.11 years, with the majority being male (25/37, 67.57%) overall. Among these patients, 14 individuals (37.84%) who received BTKi as initial therapy were categorized as treatment-naïve, while the remaining patients were R/R patients (23, 62.16%). Notably, treatment-naïve patients were significantly younger than R/R patients (56.93 ± 10.64 years vs. 66.16 ± 8.16 years, *p* = 0.005). Males comprised 78.57% in the treatment-naïve patients and 60.87% in the R/R patients, with no significant differences observed in the sex distribution between two groups. A significantly higher proportion of treatment-naïve patients had national medical insurance compared with the R/R patients (50% vs. 13.04%). Most patients were classified as Rai stage IV (54.05%, 20/37), most patients experienced cytopenias (70.27%, 26/37), and most patients were categorized as Binet stage C (64.86%, 24/37), while only 10.81% (4/37) of patients had bulky disease (≥5 cm). 43.24% (16/37) of the overall cohort had del(17p)/TP53 alterations. IGHV mutation was observed in 21.62% (8/37) of the overall cohort, including 35.71% (5/14) of treatment-naïve patients and 13.04% (3/23) of R/R patients. However, IGHV status was missing in a substantial proportion of patients, particularly among R/R patients (73.91%). The presence of del(11q) was observed in 8.11% (3/37) of patients overall, and del(13q) were noted in 5.41% (2/37) of the patients overall. No significant differences were observed between the treatment-naïve and R/R groups regarding the clinical characteristics mentioned above (Table 1). BTKi combination therapy was significantly more common in treatment-naïve patients than in R/R patients. The reasons for discontinuation included resistance (18/37, 48.65%), intolerance (12/37, 32.43%), and other factors (7/37, 18.92%) overall (Table 1, Figure 2). Among patients who discontinued BTKi due to resistance, 83.33% (15/18) had progressive disease, while 16.67% (3/18) had Richter's transformation. Among patients who discontinued BTKi due to intolerance, the main reason for intolerance was infection (6/12, 50%), followed by atrial fibrillation (2/12, 16.67%), hepatitis B virus reactivation (1/12, 8.33%), hematological toxicity (1/12, 8.33%), bleeding (1/12, 8.33%), and abdominal pain (1/12, 8.33%). Patients who discontinued BTKi due to physician or patient preference (6/7, 85.71%) and financial concerns (1/7, 14.29%) were classified as other factors. The overall median duration was 18.23 months (IQR 8.51–29.17) from BTKi initiation to the last visit. Specifically, the overall median duration from BTKi initiation to discontinuation was 5.98 months (IQR 2.04–15.57), with 48.65% (18/37) discontinuing within 6 months and 67.57% (25/37) within 12 months. Then the overall median duration from BTKi discontinuation to the last visit was 6.44 months (IQR 2.04 −15.67) (Supplementary Table 1).

**Table 1:** Baseline demographics and clinical characteristics of Chinese CLL/SLL patients who discontinued BTKi therapy.

Characteristic	Overall (*N* = 37)	Treatment- naïve^*a*^ (*N* = 14)	Relapsed/refractory^*b*^ (*N* = 23)	*p-value*
**Age at the time of BTKi discontinuation, years**				0.005^*c*^
Mean (SD)	62.67 (10.11)	56.93 (10.64)	66.16 (8.16)	
**Age group at the time of BTKi discontinuation**, ***n*** **(%)**				0.006^*d*^
≤ 65 years	21 (56.76%)	12 (85.71%)	9 (39.13%)	
>65 years	16 (43.24%)	2 (14.29%)	14 (60.87%)	
**Sex**, ***n*** **(%)**				0.306^*e*^
Male	25 (67.57%)	11 (78.57%)	14 (60.87%)	
Female	12 (32.43%)	3 (21.43%)	9 (39.13%)	
**Type of medical insurance**, ***n*** **(%)**				0.018^*e*^
Commercial insurance	0 (0.00%)	0 (0.00%)	0 (0.00%)	
National medical insurance	10 (27.03%)	7 (50.00%)	3 (13.04%)	
Out-of-pocket	6 (16.22%)	3 (21.43%)	3 (13.04%)	
Patients with missing data	21 (56.76%)	4 (28.57%)	17 (73.91%)	
**Previous cancer history (other than CLL/SLL)**, ***n*** **(%)**				>0.999^*e*^
Yes	2 (5.41%)	1 (7.14%)	1 (4.35%)	
No	35 (94.59%)	13 (92.86%)	22 (95.65%)	
**Rai stage**, ***n*** **(%)**				0.495^*e*^
I	2 (5.41%)	1 (7.14%)	1 (4.35%)	
II	6 (16.22%)	2 (14.29%)	4 (17.39%)	
III	4 (10.81%)	0 (0.00%)	4 (17.39%)	
IV	20 (54.05%)	8 (57.14%)	12 (52.17%)	
Patients with missing data	5 (13.51%)	3 (21.43%)	2 (8.70%)	
**Binet stage**, ***n*** **(%)**				0.667^*e*^
A	2 (5.41%)	1 (7.14%)	1 (4.35%)	
B	6 (16.22%)	2 (14.29%)	4 (17.39%)	
C	24 (64.86%)	8 (57.14%)	16 (69.57%)	
Patients with missing data	5 (13.51%)	3 (21.43%)	2 (8.70%)	
**Bulky disease** **≥5 cm**, ***n*** **(%)**				>0.999^*e*^
Yes	4 (10.81%)	1 (7.14%)	3 (13.04%)	
No	18 (48.65%)	7 (50.00%)	11 (47.83%)	
Patients with missing data	15 (40.54%)	6 (42.86%)	9 (39.13%)	
**Cytopenias**, ***n*** **(%)**				0.666^*e*^
Yes	26 (70.27%)	9 (64.29%)	17 (73.91%)	
No	10 (27.03%)	5 (35.71%)	5 (21.74%)	
Patients with missing data	1 (2.70%)	0 (0.00%)	1 (4.35%)	
**β2-microglobulin level group**, ***n*** **(%)**				0.132^*e*^
≤ 3.5 mg/L	8 (21.62%)	5 (35.71%)	3 (13.04%)	
>3.5 mg/L	11 (29.73%)	5 (35.71%)	6 (26.09%)	
Patients with missing data	18 (48.65%)	4 (28.57%)	14 (60.87%)	
**Leukocyte level, 10** ^**9**^ **/L**				0.360^*f*^
Patients with non-missing data	36	14	22	
Median [Q1, Q3]	7.35 [5.17, 13.93]	6.13 [4.62, 10.25]	8.32 [5.57, 41.15]	
**Platelet level, 10** ^**9**^ **/L**				0.476^*c*^
Patients with non-missing data	36	14	22	
Mean (SD)	96.53 (61.48)	87.21 (48.57)	102.45 (68.88)	
**Hemoglobin level, g/L**				0.129^*c*^
Patients with non-missing data	36	14	22	
Mean (SD)	108.81 (31.30)	118.79 (37.90)	102.45 (25.19)	
**del(17p)/TP53 mutation**, ***n*** **(%)**				0.501^*e*^
No	16 (43.24%)	8 (57.14%)	8 (34.78%)	
Yes	8 (21.62%)	2 (14.29%)	6 (26.09%)	
Patients with missing data	13 (35.14%)	4 (28.57%)	9 (39.13%)	
**IGHV mutation**, ***n*** **(%)**				0.085^*e*^
No	7 (18.92%)	4 (28.57%)	3 (13.04%)	
Yes	8 (21.62%)	5 (35.71%)	3 (13.04%)	
Patients with missing data	22 (59.46%)	5 (35.71%)	17 (73.91%)	
**del(11q)**, ***n*** **(%)**				0.265^*e*^
No	21 (56.76%)	10 (71.43%)	11 (47.83%)	
Yes	3 (8.11%)	0 (0.00%)	3 (13.04%)	
Patients with missing data	13 (35.14%)	4 (28.57%)	9 (39.13%)	
**del(13q)**, ***n*** **(%)**				0.865^*e*^
No	22 (59.46%)	9 (64.29%)	13 (56.52%)	
Yes	2 (5.41%)	1 (7.14%)	1 (4.35%)	
Patients with missing data	13 (35.14%)	4 (28.57%)	9 (39.13%)	
**BTKi regimen**, ***n*** **(%)**				0.027^*e*^
BTKi-combination	12 (32.43%)	8 (57.14%)	4 (17.39%)	
BTKi-monotherapy	25 (67.57%)	6 (42.86%)	19 (82.61%)	
**BTKi type**, ***n*** **(%)**				>0.999^*e*^
Ibrutinib	34 (91.89%)	14 (100.00%)	20 (86.96%)	
Ibrutinib and zanubrutinib	1 (2.70%)	0 (0.00%)	1 (4.35%)	
Orelabrutinib	1 (2.70%)	0 (0.00%)	1 (4.35%)	
Zanubrutinib	1 (2.70%)	0 (0.00%)	1 (4.35%)	
**Reasons for BTKi discontinuation**, ***n*** **(%)**				0.129^*e*^
Resistance to BTKi therapies	**18 (48.65%)**	**6 (42.86%)**	**12 (52.17%)**	
PD	15 (83.33%)	4 (66.67%)	11 (91.67%)	
Richter's transformation	3 (16.67%)	2 (33.33%)	1 (8.33%)	
**Intolerance to BTKi therapies**	**12 (32.43%)**	**3 (21.43%)**	**9 (39.13%)**	
Infection	6 (50.00%)	1 (33.33%)	5 (55.56%)	
Atrial fibrillation	2 (16.67%)	1 (33.33%)	1 (11.11%)	
Hepatitis B virus reactivation	1 (8.33%)	0 (0.00%)	1 (11.11%)	
Hematological toxicity	1 (8.33%)	1 (33.33%)	0 (0.00%)	
Bleeding	1 (8.33%)	0 (0.00%)	1 (11.11%)	
Abdominal pain	1 (8.33%)	0 (0.00%)	1 (11.11%)	
**Other reasons**	**7 (18.92%)**	**5 (35.71%)**	**2 (8.7%)**	
Physician or patient preference	6 (85.71%)	5 (100%)	1 (50%)	
Financial concerns	1 (14.29%)	0 (0.00%)	1 (50%)	

BTKi, Bruton's tyrosine kinase inhibitor; CLL, Chronic Lymphocytic Leukemia; L, liter; mg, milligram; Q1, 25th percentile; Q3, 75th percentile; SD, standard deviation; SLL, Small Lymphocytic Lymphoma.

^*a*^Treatment-naïve patient who received BTKi therapy and discontinued.

^*b*^Relapsed/refractory patient who received BTKi therapy and discontinued.

^*c*^Two sample t-test.

^*d*^Pearson's chi-squared test.

^*e*^Fisher's exact test.

^*f*^Wilcoxon rank sum exact test.

### 3.2 Treatment patterns

**Figure 3:**
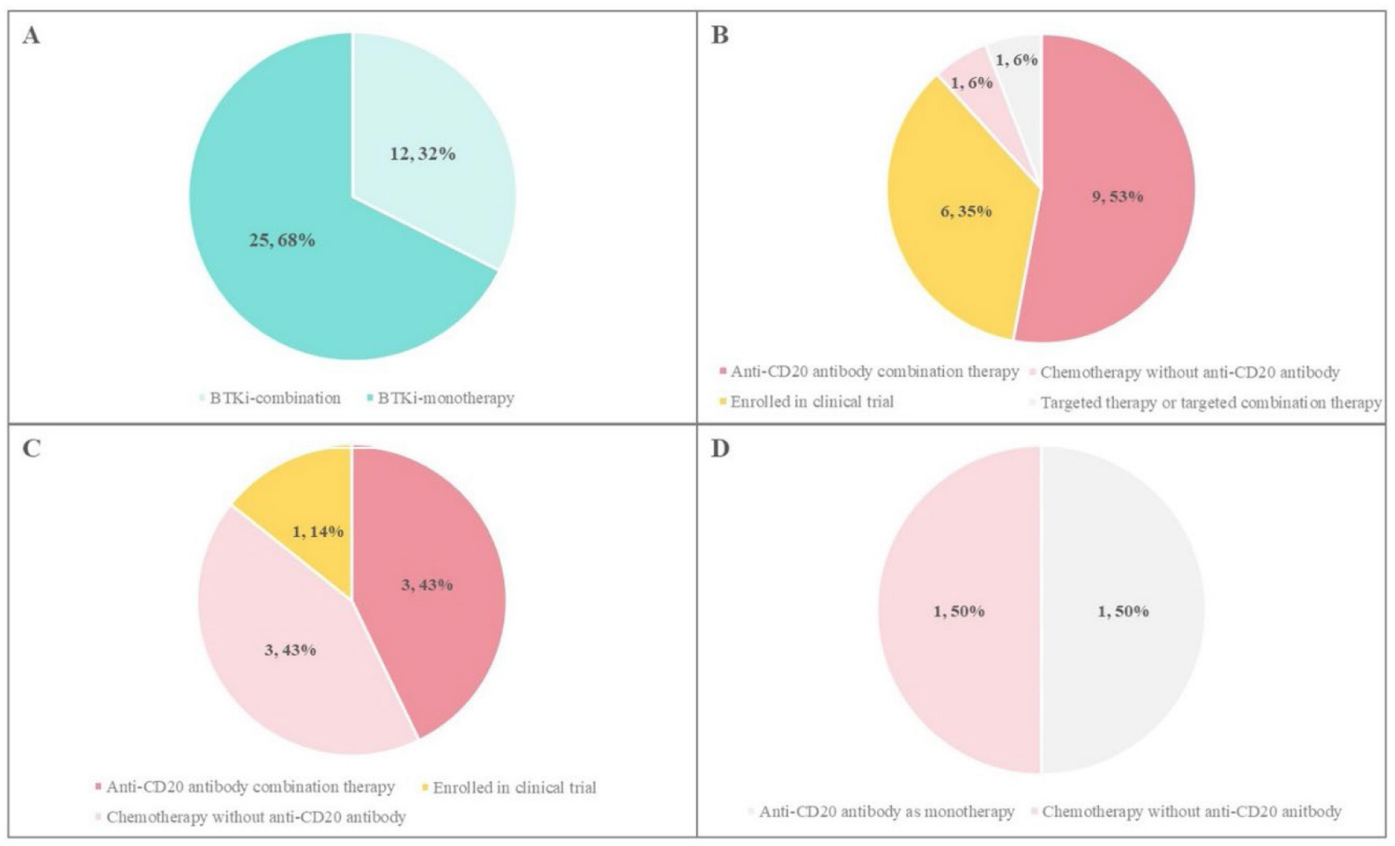
Treatment patterns of 37 patients who discontinued BTKi therapy, including distribution of BTKi regimens and subsequent therapies following BTKi discontinuation: **(A)** BTKi regimens; **(B)** first subsequent therapy after BTKi discontinuation; **(C)** second subsequent therapy after BTKi discontinuation; **(D)** third subsequent therapy after BTKi discontinuation. In each pie chart, the number before the comma indicates the number of patients receiving the corresponding treatment, and the percentage after the comma indicates the proportion within that panel-specific population.

67.57% (25/37) of overall cohort received monotherapy with BTKi (Figure 3A and Supplementary Table 2). Among treatment-naïve patients, 57.14% (8/14) opted for a combination therapy approach with BTKi, whereas 17.39% (4/23) of R/R patients utilized it. The concomitant medications used with BTKi were Rituximab, Cyclophosphamide, Vincristine, Doxorubicin, and Dexamethasone (RCHOP); Etoposide, Cyclophosphamide, Vincristine, Doxorubicin, and Dexamethasone (ECHOP); Fludarabine, Cyclophosphamide, and Rituximab (FCR); Cyclophosphamide, Chlorambucil (Supplementary Table 2). The median duration from BTKi initiation to discontinuation was 5.98 months (IQR 2.04–15.57) overall, with similar median durations observed among treatment-naïve (6.08 months, IQR 2.05–14.91) and R/R patients (5.98 months, IQR 2.21–17.09) (Supplementary Table 1). After discontinuation, the overall median duration to initiate the next treatment was 0.79 months (IQR 0.53–1.18), extending to 0.99 months (IQR 0.81–3.63) in treatment-naïve scenarios (Supplementary Figure 1). A total of 17 patients among the overall participants initiated subsequent therapy after BTKi discontinuation, followed by 7 patients transitioning to the post-BTKi second subsequent therapy, and further 2 patients commenced the post-BTKi third subsequent therapy. The most frequently used regimen was the anti-CD20 antibody combination therapy in both the post-BTKi first subsequent therapy (9/17, 52.90%) and the post-BTKi second subsequent therapy (3/7, 42.90%) (Figures 3B–D).

### 3.3 Response to BTKi treatment and outcome

**Figure 4:**
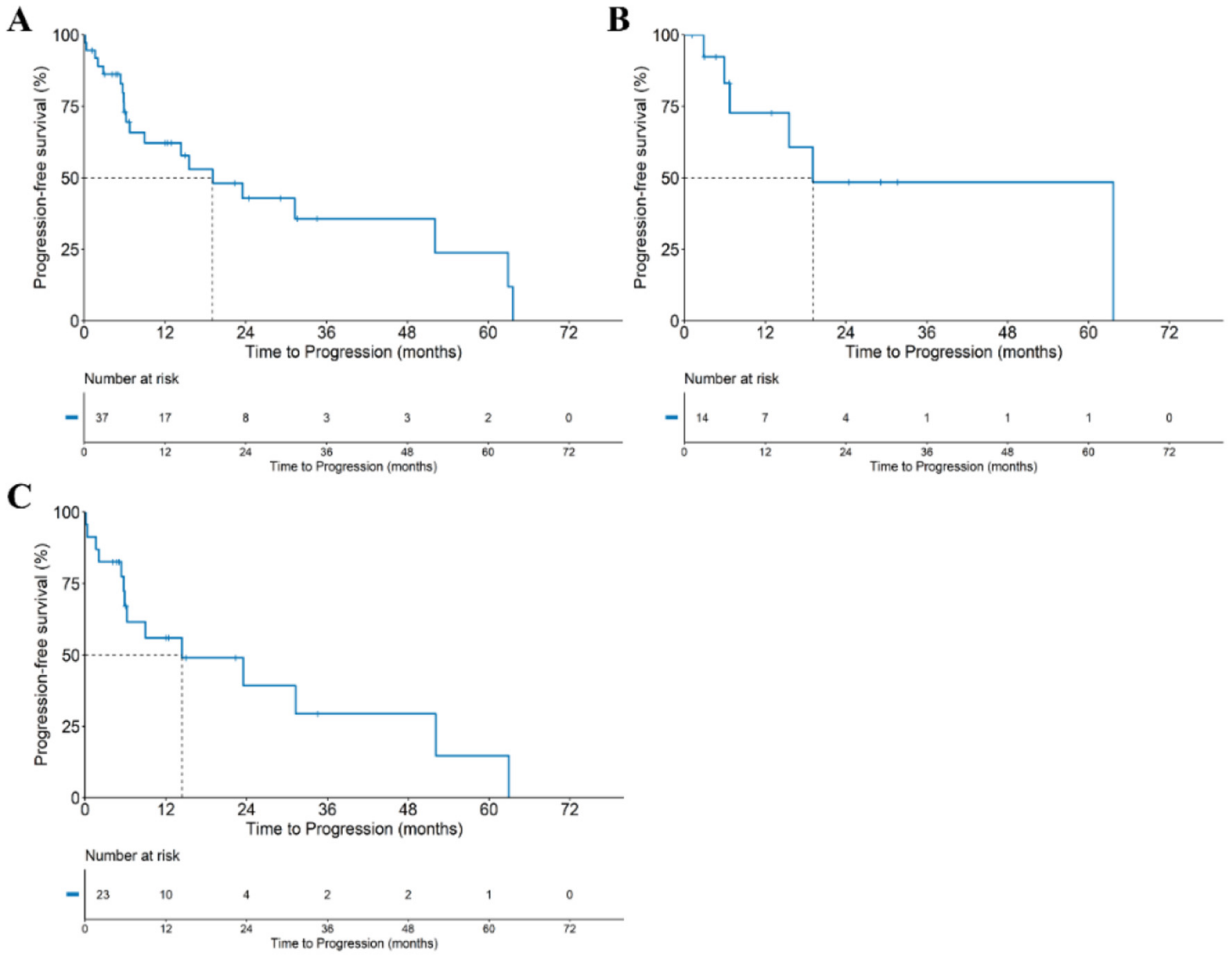
Progression-free survival (PFS) during BTKi treatment, stratified by treatment status. **(A)** Kaplan-Meier estimates of PFS for all patients; **(B)** Kaplan-Meier estimates of PFS for treatment- naïve patients; **(C)** Kaplan-Meier estimates of PFS for relapsed/refractory patients.

**Figure 5:**
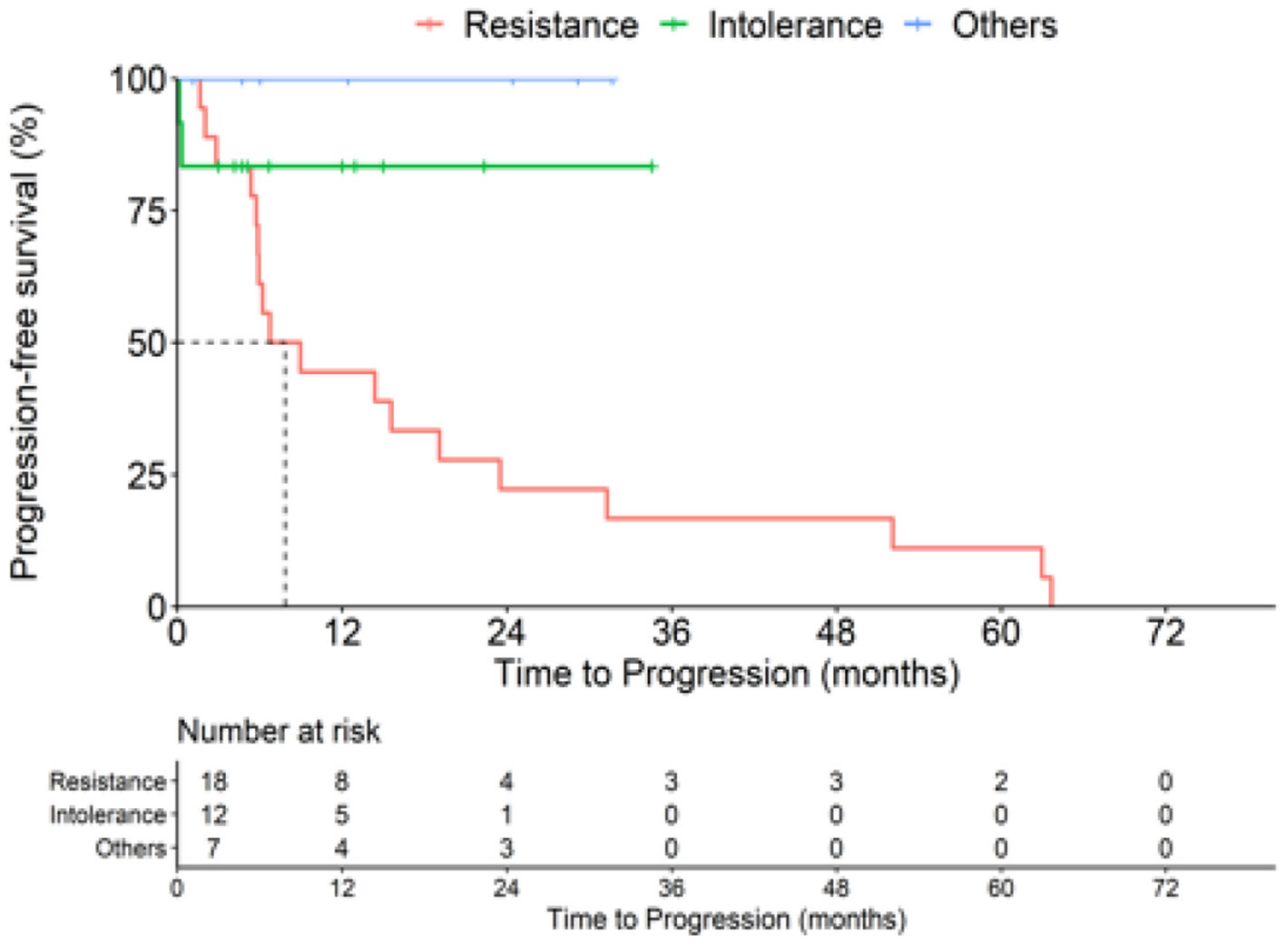
Progression-free survival during BTKi treatment, stratified by discontinuation reasons.

Among the 32 patients with available response assessment data, 25 achieved disease control, corresponding to a DCR of 78.13% following BTKi treatment. Notably, in the subgroup of treatment-naïve patients, the DCR was particularly high at 91.67%, significantly higher than that in R/R patients (70%), indicating a robust response to BTKi therapy. Regarding discontinuation reasons, patients due to resistance had the lowest DCR at 72.22%, followed by intolerance at 75.00%, and other reasons at 100% overall (Supplementary Table 3). Among the 37 patients, the median PFS during BTKi therapy in the overall population was 19.09 months (95% CI: 8.97–NR). When stratified by treatment-naïve and R/R status, the median PFS for treatment-naïve patients was 19.09 months (95% CI: 15.58–NR), while for R/R patients, it was 14.39 months (95% CI: 5.88–NR) (Figure 4). PFS during BTKi treatment was uniformly defined as the time from BTKi initiation to disease progression or death, irrespective of the subsequent reason for treatment discontinuation. Stratification by discontinuation reason was performed retrospectively to describe distinct clinical courses. When stratified by discontinuation reasons, the shortest median PFS across all subpopulations was 7.86 months (95% CI: 5.88–31.28), observed in the overall participants discontinuing due to drug resistance (Figure 5).

### 3.4 Response to post-BTKi subsequent treatment and outcome

Among the 13 patients transitioned to the first subsequent therapy after BTKi discontinuation, 11 patients experienced disease control, yielding an impressive DCR of 84.62% (Supplementary Table 3). In patients receiving second subsequent therapy after BTKi discontinuation (*n* = 6), 4 patients achieved disease control, resulting in a DCR of 66.67%. In treatment-naïve patients, the DCR was 100.00% for 6 patients on the first subsequent therapy and 66.67% for 3 patients on the second subsequent therapy, while in R/R patients, the DCR was 71.43% (5/7) and 66.67% (2/3), respectively. Regarding discontinuation reasons, 87.5% (7/8) of patients due to resistance achieved DCR on the first subsequent therapy post-BTKi treatment, and 50% (2/4) achieved DCR on the second subsequent therapy post-BTKi treatment, and 100% for patients discontinued from BTKi due to other reasons on the first subsequent therapy (4/4) and the second subsequent (2/2) therapy post-BTKi treatment (Supplementary Table 3).

**Figure 6:**
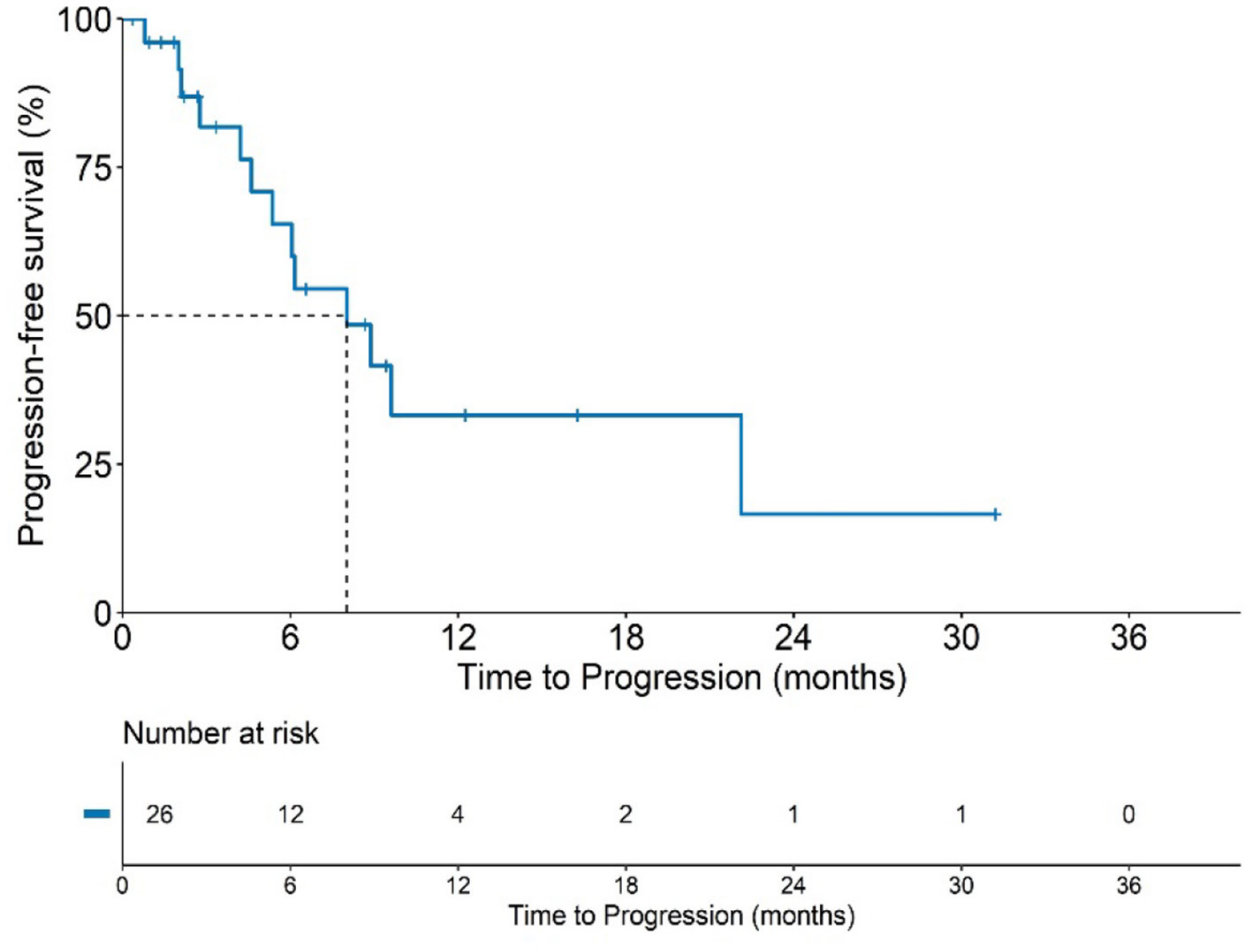
Progression-free survival (PFS) after BTKi discontinuation.

**Figure 7:**
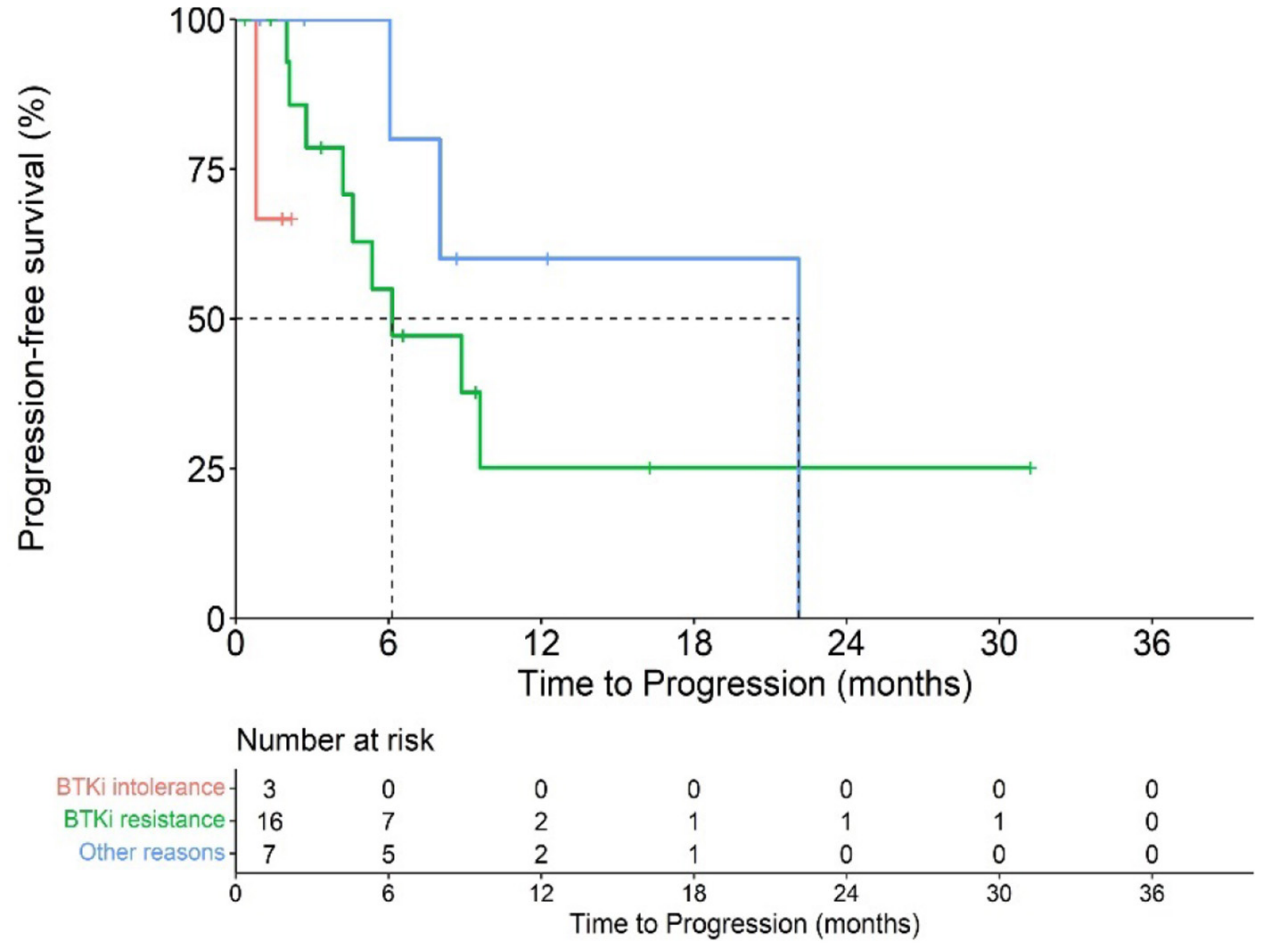
Progression-free survival for post-BTKi subsequent therapy, stratified by discontinuation reasons.

The overall median PFS for patients undergoing subsequent therapy post-BTKi discontinuation was 8.02 months (95% CI: 5.36–NR) (Figure 6). In patients receiving post-BTKi subsequent therapies, the median PFS was 8.87 months (95% CI: 6.15–NR) for the first subsequent and 5.32 months (95% CI: 4.21–NR) for the second subsequent therapies, respectively. In 7 treatment-naïve patients receiving the first subsequent therapy post-BTKi discontinuation, the overall median PFS was 15.86 months (95% CI: 8.87–NR), and for the 4 patients on the second subsequent therapy, the median was 4.60 months (95% CI: 2.00–NR), contrasting with 10 R/R patients on the first subsequent therapy who experienced a median PFS of 6.15 months (95% CI: 5.36–NR), and the median for 3 patients on the second subsequent therapy was unreached. Among patients discontinuing BTKi due to resistance, the overall median PFS for subsequent therapy was 6.15 months (95% CI: 4.60–NR), while for those discontinuing for other reasons, it was 22.12 months (95% CI: 8.02–NR). The median PFS for the intolerance subpopulation was unreached (Figure 7). It should be noted that the overall sample size and the sizes of individual subgroups were small, with a rapid depletion of the number at risk during later follow-up. As a result, the upper bounds of the confidence intervals for median PFS were not estimable (NR), particularly in the intolerance and “other reasons” subgroups, and the PFS estimates—should be interpreted with caution.

### 3.5 Survival among the participants

The overall analysis indicates that out of 37 patients, 3 deaths occurred (8.11%), with 34 patients being censored (91.89%). The median OS from BTKi discontinuation to the last record could not be determined due to insufficient events. The estimated survival probability at 6, 12, 18, and 24 months was consistently high at 0.91 (95% CI: 0.82–1) for each time point. Among treatment-naïve patients (*n* = 14), 1 death occurred (7.14%), with 13 patients censored (92.86%). The estimated survival probability at 6, 12, 18, and 24 months was consistently 0.91 (95% CI: 0.75–1) for each time point. Similarly, in the R/R group (*n* = 23), 2 deaths occurred (8.7%), with 21 patients censored (91.3%). The estimated survival probability at 6, 12, 18, and 24 months was consistent 0.91 (95% CI: 0.8–1) for each time point. No significant difference was observed in these two groups (Supplementary Figure 2).

## 4 Discussion

Our study represents the first multicenter database population-based investigation in China to examine treatment patterns and clinical outcomes among CLL/SLL patients following BTKi discontinuation in real clinical setting. As mentioned in the background, there is insufficient information regarding the discontinuation of BTKi therapy in CLL patients in China. This scarcity of data is particularly pronounced, highlighting the critical need for our study to fill these gaps and provide valuable insights into real-world clinical practices.

Through a comprehensive comparison of demographic and clinical characteristics, we identified significant differences in age, type of medical insurance and BTKi regimen between treatment-naïve patients and R/R patients who underwent BTKi discontinuation. When stratifying populations by treatment status and reasons for discontinuation, R/R patients and those discontinuing due to resistance exhibited the lowest DCR and shortest PFS among the study population. These results underscore the urgent need for effective salvage strategies for R/R patients who are unable to continue BTKi therapy, as this is essential for enhancing overall outcomes in CLL/SLL treatment.

Interestingly, the median PFS appeared identical between the overall cohort and the treatment-naïve subgroup. The identical median PFS observed for the overall cohort and the treatment-naïve subgroup may be attributable to a similar event distribution and censoring pattern in this small observational study, rather than indicating true equivalence in treatment effect between subgroups.

It is noted that most participants included in our study were R/R patients. This could be attributed to two reasons.

1. BTK inhibitors are highly effective therapies for R/R CLL in China currently, with second-generation BTKi's often preferred due to their reduced toxicity (27). Recommendations for BTKi therapy in R/R patients further support this trend (28), contributing to the higher utilization of BTKi among R/R patients compared to treatment-naïve patients and potentially resulting in a higher rate of discontinuation among the former group.

2. The high cost of BTKi therapy may serve as a barrier to their widespread usage, especially for patients lacking insurance coverage or reimbursement programs (29). These findings align with previous studies conducted in Sweden, seven Latin American countries (30, 31), and a study involving CLL patients in Jiangsu Province from November 1991 to December 2016 (32). The difference in treatment selection between treatment-naïve and R/R patients may be attributed to substantial disparities in national insurance coverage, with treatment-naïve patients generally having higher coverage compared to R/R patients. Consequently, R/R patients may experience delays in accessing suitable therapy, potentially leading to eventual discontinuation.

R/R CLL/SLL patients tend to have inferior PFS outcomes compared to treatment-naïve patients during BTKi treatment. In R/R CLL/SLL, a proportion of patients at our study sites had prior treatment experience, indicating a history of treatment failure with conventional approaches and prompting the search for newer BTKi therapies (33). This observation is consistent with findings from a clinical trial indicating that most patients with R/R CLL who discontinued BTKi therapy faced challenges in subsequent therapy and had poor outcomes (34). Notably, patients with resistance to BTKi exhibited the shortest PFS durations. Study suggests that resistance to covalent BTKi may develop over time due to mutations in BTK and downstream kinases (35). Given the continuous use of BTK inhibitors, the emergence of drug resistance is inevitable, leading to loss of response or disease progression (36). Therefore, comprehending the mechanisms of resistance and implementing sensitive detection methods for emergent resistant clones during therapy is crucial for guiding the development of new therapies, therapeutic sequencing, and rational drug combinations in the future.

Our study brings innovation by focusing on DCR and PFS across multiple drug usage after the discontinuation of BTKi therapy. This approach explores the potential adverse outcomes associated with BTKi discontinuation among Chinese patients. From a global perspective, there are already some innovative drugs and therapies, such as non-covalent BTKi, B-cell lymphoma-2 inhibitors (BCL2i), and other new small molecule targeted therapies, which can overcome the challenge of BTKi treatment failure (37). However, in China, none of these drugs have been approved for CLL/SLL patients. In the study, the most frequently utilized regimen was the anti-CD20 antibody combination therapy, both in the post-BTKi first subsequent and second subsequent therapies, which showed relatively poor clinical outcomes. Also, considering the impact of the patient's own condition and personal economic factors, in our study in China, different patients receive differentiated treatment regimens in real clinical practice (Supplementary Table 2). These suggest that in actual clinical practice in China, the accessibility and choices of novel therapies after BTKi treatment failure are limited compared to other countries (38), highlighting the urgent need for expediting new drug approvals for CLL/SLL Chinese patients discontinuing BTKi therapies. It is noteworthy that limited studies have specifically assessed clinical outcomes post-BTKi treatment, posing challenges in making direct comparisons. A previous study analyzing OS after BTKi discontinuation reported a median survival of 20.6 months (39). Furthermore, an estimated PFS rate at 12 months, defined as the duration from BTKi initiation to death or disease progression, was reported as 86.3% (95% CI: 81.3%−91.2%) in Denmark (19). In this study, compared to the PFS with BTKi treatment, the PFS after discontinuation of BTKi was relatively shorter. Early discontinuation of BTKi therapy has been associated with poor overall outcomes, particularly for those discontinuing due to disease progression, consistent with our results. It is important to acknowledge that demonstrated PFS may be influenced by the duration of observation, as longer periods allow for the observation of more clinical outcomes. Meanwhile, the results showed that BTKi therapy in combination was more common in the treatment-naïve population, which may have also influenced the PFS results. Additionally, previous study has reported suboptimal outcomes for patients discontinuing BTKi regimens, especially those experiencing CLL progression or Richter transformation (34). This study observed inferior outcomes for patients who experienced BTKi discontinuation and initiated subsequent therapies, further supporting the conclusions of the aforementioned study. However, establishing a cause-effect relationship between BTKi discontinuation and clinical outcomes requires further investigation.

Several limitations should be noted in our study. Firstly, the EMR data used in our analysis were extracted from three Grade-A tertiary hospitals located in northeast China. This may restrict the representativeness of our findings and limit their generalizability to the entire Chinese population, despite one of the hospitals being recognized as the best hematology specialized Grade-A tertiary hospital in China. Secondly, our data collection was retrospective rather than prospective, which introduces the possibility of missing information that could potentially affect certain results, especially time-dependent endpoints such as PFS. To address this concern, we performed imputation and sensitivity analyses to minimize or evaluate the potential bias in the results. Thirdly, the overall length of observation period in our study may still not be long enough to fully capture treatment patterns, particularly in later treatments after BTKi discontinuation. Lastly, it is important to note that our study relied on real-world measurements from clinical practice rather than data from controlled clinical trials. This difference in data source could introduce variability and potential confounding factors. Despite these limitations, our comprehensive study provides valuable evidence that can contribute to understanding the real-world unmet medical needs for CLL/SLL patients who have discontinued BTKi therapy and the urgency of expediting new drug approvals for these patients.

Our study presents reasons for BTKi discontinuation, post-BTKi treatment strategies, and subsequent therapy outcomes among CLL/SLL patients in real-world clinical practice in China. These results show the influence of treatment status and reasons for BTKi discontinuation on the outcomes of subsequent therapy, offering valuable insights into the dynamics of disease control in this patient population. Additionally, the study results also indicate the urgency of expediting new drug approvals for CLL/SLL patients in China.

## Data Availability

The datasets used and/or analyzed during the current study are available from the corresponding author on reasonable request.
