# Supplementary material for: Disease characteristics, treatment, and outcomes in Chinese chronic lymphocytic leukemia patients following BTK inhibitor discontinuation: a multicenter real-world study
Source: Front Med (Lausanne). 2026 Jun 12;13:1739102. doi: 10.3389/fmed.2026.1739102 (PMC13307203; doi:10.3389/fmed.2026.1739102)
Supplement: Supplementary file 1 [file Data_Sheet_1.docx]

Supplementary Material

# Supplementary Tables

**Supplementary Table 1. Observation periods of Chinese CLL/SLL patients who discontinued BTKi therapy**

| **Characteristic** | **Overall (N = 37)** | **Treatment-naïve^1^ (N = 14)** | **Relapsed/refractory^2^ (N = 23)** | **P-value** |
| --- | --- | --- | --- | --- |
| **Duration from initial diagnosis to BTKi initiation^3^, months** |  |  |  | <0.001^4^ |
| N | 37 | 14 | 23 |  |
| Mean (SD) | 35.66 (30.32) | 16.33 (16.73) | 47.43 (30.92) |  |
| Median [Q1, Q3] | 29.17 [9.07, 51.71] | 12.74 [1.99, 24.76] | 47.61 [26.37, 60.29] |  |
| Min, max | 0.95, 105.30 | 0.95, 57.59 | 1.18, 105.30 |  |
| **Duration from BTKi initiation to discontinuation^5^, months** |  |  |  | 0.814^6^ |
| N | 37 | 14 | 23 |  |
| Mean (SD) | 12.85 (16.53) | 11.96 (16.75) | 13.39 (16.75) |  |
| Median [Q1, Q3] | 5.98 [2.04, 15.57] | 6.08 [2.05, 14.91] | 5.98 [2.21, 17.09] |  |
| Min, max | 0.10, 63.64 | 0.20, 63.64 | 0.10, 62.95 |  |
| **Duration from BTKi discontinuation to initiation of sequent treatment^7^, months** |  |  |  | 0.204^6^ |
| N | 17 | 7 | 10 |  |
| Mean (SD) | 2.32 (3.94) | 3.54 (5.23) | 1.46 (2.71) |  |
| Median [Q1, Q3] | 0.79 [0.53, 1.18] | 0.99 [0.81, 3.63] | 0.63 [0.43, 0.99] |  |
| Min, max | 0.03, 14.88 | 0.03, 14.88 | 0.16, 9.13 |  |
| **Duration from BTKi initiation to last visit^8^, months** |  |  |  | 0.506^4^ |
| N | 37 | 14 | 23 |  |
| Mean (SD) | 21.86 (18.64) | 22.91 (16.20) | 21.21 (20.30) |  |
| Median [Q1, Q3] | 18.23 [8.51, 29.17] | 23.95 [12.30, 31.05] | 17.77 [6.61, 28.41] |  |
| Min, max | 0.13, 78.59 | 2.83, 63.64 | 0.13, 78.59 |  |
| **Duration from BTKi discontinuation to last visit^9^, months** |  |  |  | 0.481^6^ |
| N | 37 | 14 | 23 |  |
| Mean (SD) | 9.04 (9.22) | 10.98 (10.76) | 7.86 (8.18) |  |
| Median [Q1, Q3] | 6.44 [2.04, 15.67] | 7.76 [2.26, 16.09] | 3.84 [2.32, 14.21] |  |
| Min, max | 0.03, 32.26 | 0.03, 32.26 | 0.03, 31.34 |  |

Abbreviations: BTKi = Bruton’s tyrosine kinase inhibitor; PD = Progression of disease; Q1 = 25th percentile; Q3 = 75th percentile; SD = standard deviation.

Notes:

1. Treatment-naïve patient who received BTKi therapy. Treatment-naïve was defined as patients who received BTKi as initial therapy when enrolled into the study.
2. Relapsed/refractory patient who received BTKi therapy
3. Time from initial diagnosis of CLL/SLL to BTKi initiation = (Initial diagnosis date –Initiation date of BTKi therapy + 1)/ 30.4375.
4. Wilcoxon rank sum exact test
5. Time from BTKi initiation to discontinuation = (The date of BTKi discontinuation - Initiation date of BTKi therapy + 1)/ 30.4375.
6. Wilcoxon rank sum test
7. Time from BTKi discontinuation to initiation of sequent treatment = (Initiation date of sequent treatment - The date of BTKi discontinuation + 1)/ 30.4375.
8. Time from BTKi initiation to last visit = (The date of last visit - The date of BTKi initiation + 1)/ 30.4375.
9. Time from BTKi discontinuation to last visit = (The date of last visit - The date of BTKi discontinuation + 1)/ 30.4375.

**Supplementary Table 2. Duration of treatment during BTKi therapy and post-BTKi Therapy**

| **Line of therapy** | **Number of patients** | **Percentage** | **Number of patients with treatment discontinuation** | **Median DoT^1^ (95%CI), months** | |
| --- | --- | --- | --- | --- | --- |
| **BTKi regimen** | 37 | 100.00% | 37 |  | |
| BTKi-combination (iFCR n=9, iRCHOP n=2, iECHOP n=1) | 12 | 32.43% | 12 | 4.83(2.37, -) | |
| BTKi-monotherapy | 25 | 67.57% | 25 | 6.47(5.32, 22.25) | |
| **First-line** **therapy after BTKi discontinuation** | 17 | 100.00% |  |  | |
| Anti-CD20 antibody combination therapy (BR n=3, RCHOP n=3, RGDP n=2, REDOCH n=1) | 9 | 52.90% | 6 | 7.16(3.68, -) |  |
| Chemotherapy without anti-CD20 antibody (FC n=1) | 1 | 5.90% | 0 | -(-, -) | |
| Targeted therapy or targeted combination therapy (B+V n=1) | 1 | 5.90% | 1 | 0.03(-, -) | |
| Enrolled in clinical trial | 6 | 35.30% | 4 | 4.66(2.73, -) | |
| **Second-line therapy after BTKi discontinuation** | 7 | 100.00% |  |  | |
| Anti-CD20 antibody combination therapy (BR2 n=1, R2 n=1, RCHOP n=1) | 3 | 42.90% | 3 | 3.22(1.15, -) | |
| Chemotherapy without anti-CD20 antibody (ESHAP n=1, B n=1, Len n=1) | 3 | 42.90% | 1 | 1.05(-, -) | |
| Enrolled in clinical trial | 1 | 14.30% | 0 | -(-, -) | |
| **Third-line therapy after BTKi discontinuation** | 2 | 100.00% |  |  | |
| Anti-CD20 antibody as monotherapy (R n=1) | 1 | 50.00% | 0 | -(-, -) | |
| Chemotherapy without anti-CD20 antibody (ECHOP n=1) | 1 | 50.00% | 0 | -(-, -) | |

Abbreviations: BTKi = Bruton’s tyrosine kinase inhibitor; CD20 = cyclophosphamide+vincristine+doxorubicin+dexamethasone; CI = confidence interval; DoT= duration of treatment, -/NR = Not reached; n = Number of patient; i = Ibrutinib, FCR = Fludarabine, Cyclophosphamide and Rituximab; RCHOP = Rituximab, Cyclophosphamide, Vincristine, Doxorubicin and Dexamethasone, ECHOP = Etoposide, Cyclophosphamide, Vincristine, Doxorubicin and Dexamethasone; BR = Bendamustine, Rituximab; RGDP = Rituximab, Gemcitabine, Dexamethasone, and Cisplatin; REDOCH = Rituximab, Etoposide, Dexamethasone, Vincristine, Cyclophosphamide, Liposomal Doxorubicin; FC = Fludarabine, Cyclophosphamide, B = Bendamustine; V = Venetoclax; BR2 = Bendamustine, Rituximab, Lenalidomide; R2 = Rituximab, Lenalidomide; ESHAP = Etoposide, Methylprednisolone, high-dose Cytarabine, and Cisplatin; Len = Lenalidomide; R = Rituximab

**Supplementary Table 3. Disease Control Rate (DCR) of BTKi Therapy and post-BTKi therapy stratified by Treatment Status and discontinuation reasons**

| **Line of therapy** | **Number of evaluable patients, n** | **Number of patients with disease control (CR + PR + Stable disease), n** | **DCR^1^ (%)** |
| --- | --- | --- | --- |
| **DCR by treatment status** | | | |
| **DCR, overall** |  |  |  |
| BTKi | 32 | 25 | 78.13% |
| First therapy after BTKi discontinuation | 13 | 11 | 84.62% |
| Second therapy after BTKi discontinuation | 6 | 4 | 66.67% |
| **DCR, Treatment-naïve** |  |  |  |
| BTKi | 12 | 11 | 91.67% |
| First therapy after BTKi discontinuation | 6 | 6 | 100.00% |
| Second therapy after BTKi discontinuation | 3 | 2 | 66.67% |
| **DCR, Relapsed/refractory** |  |  |  |
| BTKi | 20 | 14 | 70.00% |
| First therapy after BTKi discontinuation | 7 | 5 | 71.43% |
| Second therapy after BTKi discontinuation | 3 | 2 | 66.67% |
| **DCR by discontinuation reasons** | | | |
| **DCR, Resistance to BTKi therapies** |  |  |  |
| BTKi | 18 | 13 | 72.22% |
| First therapy after BTKi discontinuation | 8 | 7 | 87.50% |
| Second therapy after BTKi discontinuation | 4 | 2 | 50.00% |
| **DCR, Intolerance to BTKi therapies** |  |  |  |
| BTKi | 8 | 6 | 75.00% |
| First therapy after BTKi discontinuation | 1 | 0 | 0.00% |
| Second therapy after BTKi discontinuation | / | / | / |
| **DCR, Other reasons** |  |  |  |
| BTKi | 6 | 6 | 100.00% |
| First therapy after BTKi discontinuation | 4 | 4 | 100.00% |
| Second therapy after BTKi discontinuation | 2 | 2 | 100.00% |

Abbreviations: BTKi = Bruton’s tyrosine kinase inhibitor; CR = Complete remission; DCR = Disease control rate; PD = Progression of disease; PR= Partial remission

Note:

1.DCR = (total number of patients with CR, PR or SD) / total number of the evaluable population *100%.

# Supplementary Figures


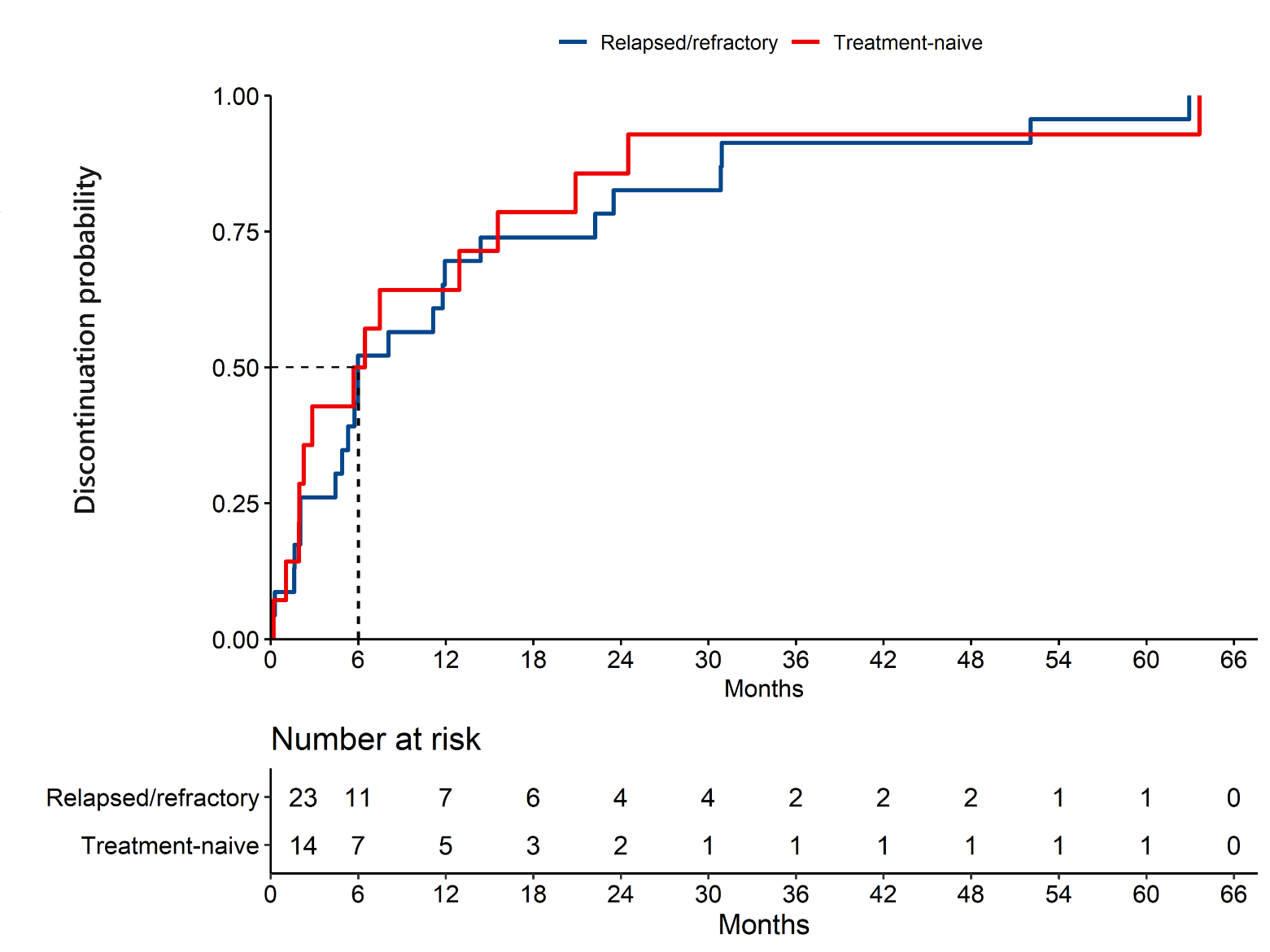


**Supplementary Figure 1. Kaplan-Meier curve for BTKi treatment initiation to discontinuation**


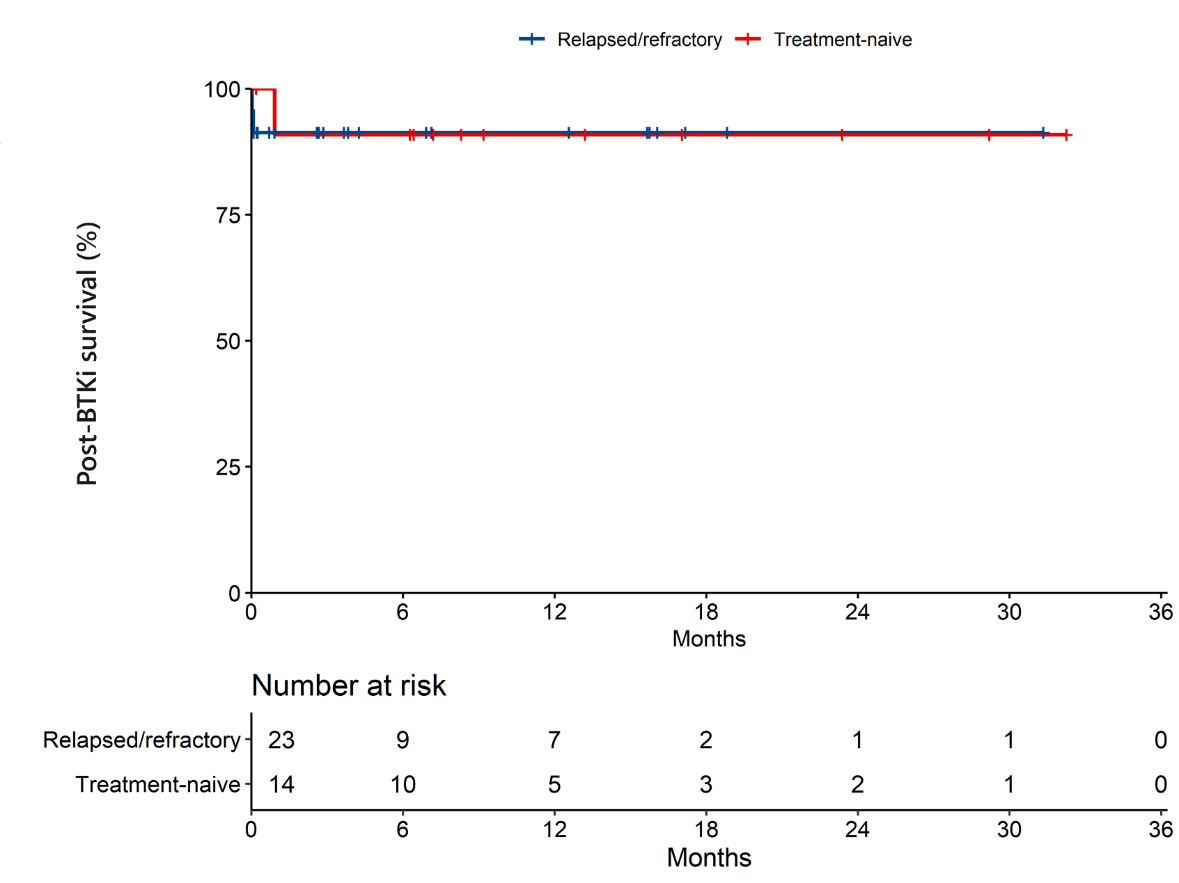


| **Group** | **Number of patients** | **Death^1^, n (%)** | **Censor, n (%)** | **Post-BTKi survival rate at 6 Months**  **(95% CI)** | **Post-BTKi survival rate at 12 Months**  **(95% CI)** | **Post-BTKi survival rate at 18 Months**  **(95% CI)** | **Post-BTKi survival rate at 24 Months**  **(95% CI)** |
| --- | --- | --- | --- | --- | --- | --- | --- |
| Overall | 37 | 3 (8.11) | 34 (91.89) | 0.91 (0.82, 1) | 0.91 (0.82, 1) | 0.91 (0.82, 1) | 0.91 (0.82, 1) |
| Treatment-naïve | 14 | 1 (7.14) | 13 (92.86) | 0.91 (0.75, 1) | 0.91 (0.75, 1) | 0.91 (0.75, 1) | 0.91 (0.75, 1) |
| Relapsed/refractory | 23 | 2 (8.7) | 21 (91.3) | 0.91 (0.8, 1) | 0.91 (0.8, 1) | 0.91 (0.8, 1) | 0.91 (0.8, 1) |

Abbreviations: BTKi = Bruton’s tyrosine kinase inhibitor; CI = confidence interval; OS=Overall survival.

Note:

1. Death is defined as confirmed death.

**Supplementary Figure 2. Post-BTKi survival according to treatment status**
